# CTSK as a Collagen Degradation Marker Induces Gingival Recession During High-Force Orthodontic Tooth Movement

**DOI:** 10.1016/j.identj.2025.03.019

**Published:** 2025-05-01

**Authors:** Yanfang Wang, Yili Xu, Yutong Lu, Xinying Su, Yonghua Lei

**Affiliations:** a Department of Orthodontics, Center of Stomatology, Xiangya Hospital, Central South University, Changsha, China; b Department of Stomatology, Liuzhou People's Hospital, Liuzhou, China

**Keywords:** Gingival recession, Orthodontic tooth movement, Cathepsin K, Periodontitis

## Abstract

**Introduction and aims:**

Gingival recession is a common complication of orthodontic treatment that affects both aesthetics and periodontal health. While traditionally associated with bone resorption, recent research suggests that Cathepsin K (CTSK) play a significant role in collagen fibre degradation within periodontal connective tissues. This study combines animal experiments and clinical research to investigate whether CTSK plays a role in the process of gingival recession during orthodontic tooth movement (OTM).

**Methods:**

An OTM model was created using the maxillary first molar in mice. Differences in gingival tissue thickness and height between experimental and control groups were statistically analysed. Additionally, in the clinical study, CTSK expression in gingival crevicular fluid (GCF) was assessed. CTSK mRNA expression in gingival crevicular fluid was evaluated in orthodontic patients, comparing healthy and gingival recession groups.

**Results:**

High-force OTM significantly decreased the thickness and height of mesial gingival tissues (*P* < .005). In the gingival recession group, the number of cells within the region of interest (ROI) decreased, while the number of CTSK+ cells increased significantly (*P* < .0005). RT-qPCR analysis showed that CTSK mRNA expression in GCF of gingival recession patients was significantly higher than in the control group (*P* < .05).

**Conclusion:**

High-force orthodontic tooth movement induced gingival recession in mice. The results of the animal experiment suggested that CTSK contributes to collagen fibre degradation in gingival connective tissue, leading to recession. Studies of human GCF have further supported the role of CTSK as a marker of collagen degradation in gingival recession.

**Clinical relevance:**

These findings may offer new insights for the clinical management of complications such as "black triangles" following orthodontic treatment.

## Introduction

Orthodontic tooth movement (OTM) involves the remodelling of the alveolar bone and periodontal ligament.^1^ As teeth move, both hard and soft periodontal tissues undergo adaptive changes, characterized by bone resorption on the pressure side and bone formation on the tension side, along with remodelling of the periodontal ligament fibres and reconstruction of the gingival morphology. The results and success of orthodontic treatment depend on whether the periodontal tissues remain stable and undamaged, without excessive destruction or irreversible injury, as teeth move to their new positions.^2^ Gingival recession is a common soft tissue abnormality during orthodontic treatment, manifesting as the free gingival margin moving away from the enamel-cementum junction and migrating towards the root.^3^ When recession occurs at the gingival papilla, a “black triangle” can form between two teeth, severely impacting the aesthetic appearance of the anterior teeth area.^4^ Gingival recession not only leads to poor aesthetic effects but also increases root sensitivity and susceptibility to caries lesions.^2^

Current research on the mechanisms of gingival recession primarily focuses on the biological level. When excessive orthodontic forces cause tooth movement beyond the physiological limits of the alveolar ridge, the position of the tooth root may approach or exceed the outer cortical plate of the alveolar bone, leading to rapid resorption of the hard tissue under pressure.^2^^,^^5^ This loss of bone tissue can lead to the formation of fenestrations or dehiscences. Due to the lack of alveolar bone support, the free gingival margin will migrate towards the root along the bone level at the cervical region.^2^^,^^3^^,^^5^, ^6^, ^7^, ^8^ There is currently a lack of in-depth exploration of the molecular mechanisms involved in the process of gingival recession. The main characteristic of gingival recession is the degradation of connective tissue, particularly the breakdown of collagen fibres. Typically, 3 major categories of enzymes are closely related to the degradation of the connective tissue matrix: the matrix metalloproteinases family, the cysteine protease family, and the acid phosphatase family. Cathepsin K, a member of the cysteine protease family, exhibits strong collagenase activity within the pH range of 4.0 to 7.0, even surpassing that of matrix metalloproteinases-1, -9, and -13.^9^ This high collagenase activity makes CTSK a novel marker for collagen fibre degradation, particularly in collagen degradation such as gingival recession.

Therefore, the objectives of this study are: (1) to establish an animal model of OTM gingival recession in mice; (2) to investigate whether this gingival recession process is associated with CTSK, thereby revealing its role in the degradation of connective tissue at the molecular level.

## Material and methods

### Single-cell RNA-seq analysis and RNA-seq analysis

**Fig. 1:**
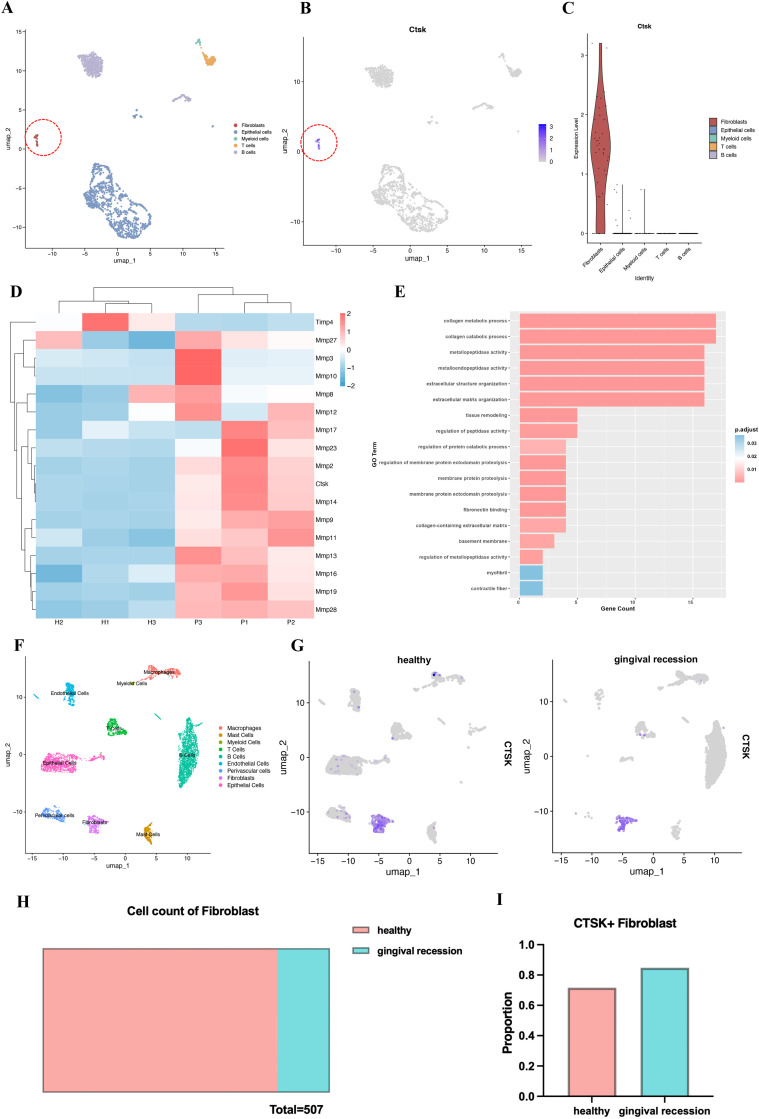
scRNA-seq (GSE201108) shows Cathepsin K (Ctsk) expression in gingival tissue of mice with early periodontitis: A, UMAP clustering of various cell populations. B, FeaturePlot showing the distribution of Ctsk expression across different cell populations. C, VlnPlot showing the expression levels of Ctsk across different cell types. D-E, RNA-seq (GSE186882) illustrates the heatmap of differential gene expression and the results of GO pathway enrichment analysis between gingival tissue of mice with periodontitis and healthy gingival tissue. scRNA-seq (GSE152042) shows Cathepsin K (Ctsk) expression in gingival tissue of human: F, the UMAP clustering of various cell populations. G, FeaturePlot showing the distribution of Ctsk expression in the healthy group and the gingival recession group. H, Comparison of the number of fibroblasts between the healthy and periodontitis groups. I, Comparison of the proportion of CTSK+ fibroblasts between the healthy and periodontitis groups.

The scRNA-seq dataset GSE201108 of gingival tissues from mice with early-stage periodontitis was downloaded from the GEO database and analysed using the R package Seurat for single-cell genomics.^10^ Quality control was performed according to the original method, followed by dimensionality reduction using the FindVariableFeatures, ScaleData, and RunPCA functions. The FindNeighbors and FindClusters functions were used for clustering, and the RunUMAP function was applied for data visualization. Five different cell types were annotated based on the original classification method (Figure 1A): B cells (Cd19), T cells (Cd3e), Myeloid cells (Lyz2), Epithelial cells (Krt5), and Fibroblasts (Col1a1). The distribution of Ctsk gene expression in the UMAP plot of gingival tissues from mice with early-stage periodontitis was analysed, and the split.by function was used to group the samples by cell type to display and compare CTSK gene expression.

The RNA-seq dataset GSE186882, consisting of gingival tissues from healthy mice and mice with periodontitis, was downloaded from the GEO database.^11^ After data filtering and normalization, differential expression analysis was performed using the DESeq2 package. The criteria for selecting differentially expressed genes were an adjusted *P* value of less than .05 and |log2FoldChange| greater than 1. A heatmap of the expression levels of the selected differentially expressed genes across different samples was generated using the pheatmap package, and Gene Ontology (GO) enrichment analysis of the differentially expressed genes was conducted using the clusterProfiler package.

The scRNA-seq dataset GSE152042, consisting of gingival tissues isolated from healthy individuals and periodontitis patients, was downloaded from the GEO database.^12^ The data from the "Healthy1", "Healthy2", and "Serve" groups were imported into RStudio and labelled as the "healthy" and "periodontitis" groups. Analysis was performed using the Seurat package, following the methods described in the original text for data quality control, data scaling, dimensionality reduction, clustering, and visualization. According to the cell markers described in the original text, 9 different cell types were annotated (Figure 1F): Epithelial cells (HOPX, IGFBP5, LAMB3, KRT1, KRT8, LAT, PTGER, MKI67, and TOP2A); Fibroblasts (COL1A1 and COL3A1); Perivascular cells (PDGFRB and RGS5); Endothelial cells (CLDN5, EMCN, KDR, TIE1, and SOX18); B cells (MZB1, DERL3, IGHG4, MS4A1, and CD37); T cells (CXCR6); Myeloid cells (CLEC9A and IRF8); Mast cells (TPSB2 and TPSB1); Macrophages (LYZ and AIF1). The expression of the CTSK gene in the "healthy" and "periodontitis" groups was compared using the split.by function. The numbers and proportions of CTSK+ fibroblasts in the "healthy" and "periodontitis" groups were statistically analysed and visualized.

### Animal experiment

This experiment was approved by the Ethics Committee of Xiangya Hospital, Central South University. All animal experiments complied with the ARRIVE guidelines.

Male C57BL/6 mice, aged 10 weeks and in good health, were purchased from Hunan Slike Jingda Company(n = 9). All mice were of the specific pathogen free (SPF) grade and were housed at the Animal Experiment Center of Xiangya School of Medicine, Central South University. The environmental conditions included controlled temperature (25 °C), humidity (65%), and maintained under climate controlled conditions with a 12 h light/dark cycle and fed with standard chow and drinking water ad libitum.

A mouse model of OTM was constructed. Mice were anesthetized via intraperitoneal injection of 1% sodium pentobarbital and fixed in a supine position on the surgical board, with the oral cavity exposed using a custom-made mouth opener. One side of the mouse's maxillary first molar (M1) and the maxillary incisors were etched. A Niti open coil spring was cut to 3 mm, and both ends were extended using ligature wire. One end was bonded to the surface of M1, and the other end was stretched with an orthodontic force gauge and bonded to the incisors. The mice were divided into two groups: the light-force group with a force of 30 cN and the high-force group with a force of 90 cN. The contralateral maxillary first molar was left untreated as an internal control. Post-surgery, mice were fed soft food, and the fixation of the Niti open coil spring was checked daily.

To investigate the role of CTSK in gingival recession, we used odanacatib (ODN), a CTSK inhibitor.^13^ Two groups of high-force OTM mouse models were established. Mice in the experimental group were orally administered 3.606 mg/kg/week of pharmacologic grade ODN (Selleck, USA, MK-0822) in dimethylsulfoxide (DMSO) from 1 week before OTM model establishment until sample collection. The control group received oral administration of DMSO.^14^

After 7 days of orthodontic force application, the mice were sacrificed by cervical dislocation, and the maxillary bones were harvested. The force appliance was removed, and the maxillary bones were fixed, paraffin-embedded, sectioned, and stored at room temperature.

### Histological staining and quantitative image analysis

**Fig. 2:**
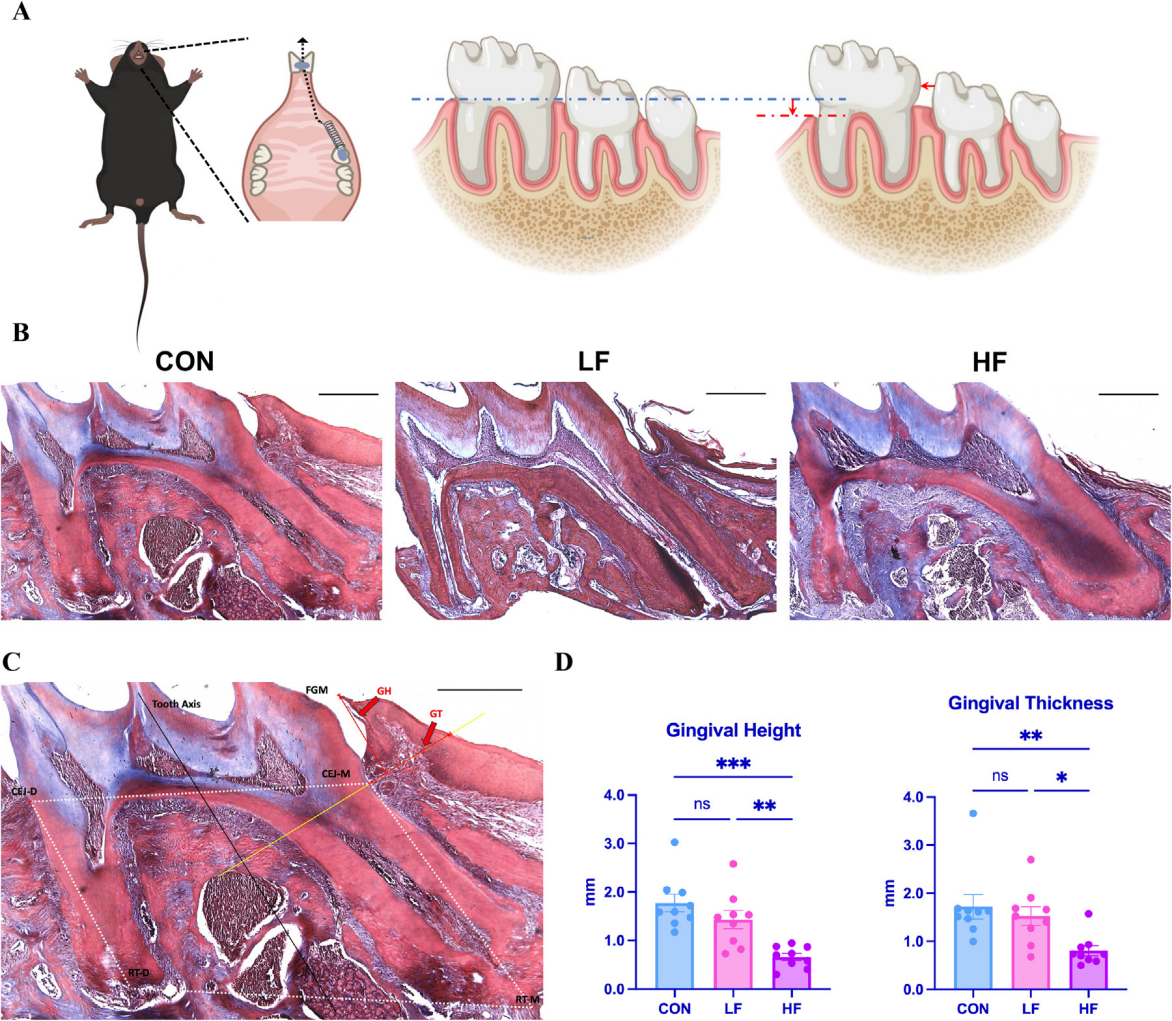
A, Construction of a mouse model of gingival recession; B, Gingival morphology of the control group (CON) and experimental group (LF and HF); C, Measurement of gingival height (GH) and gingival thickness (GT) in the mesial gingival region of the first molar in mice; D, Statistical analysis of gingival height (GH) and gingival thickness (GT) in the mesial gingival region of the first molar among the control group (CON) and experimental groups (LF and HF) was performed using a 1-way ANOVA test (n = 9/group, *P < .05,**P < .01, ***P < .001).

Tissue sections were subjected to Masson's trichrome staining (Solarbio, Beijing), and the samples were observed and imaged using an upright fluorescence microscope (Leica, DM4B). Gingival height (GH) and gingival thickness (GT) were assessed following the method of Danz JC et al (Figure 2C).^5^ First, the following anatomical landmarks were identified: free gingival margin (FGM);mesial cement-enamel junction (CEJ-M);distal cement-enamel junction (CEJ-D);mesial root tip (RT-M); distal root tip (RT-D).Then, interactive geometrical constructions were defined: tooth axis (TA) as the apico-coronal bisector of a tetragon with CEJ-D, CEJ-M, RT-D and RT-M as corner points.Finally, the following parameters were evaluated: Gingival height (GH) as the shortest distance from FGM to a perpendicular line to TA going through CEJ-M; gingival thickness (GT) is defined as the distance from the intersection of the perpendicular line to the tooth's long axis at the mesial cementoenamel junction (CEJ-M) to the most distant point of the gingiva.

### Immunofluorescence staining

Tissue sections were deparaffinized and rehydrated in xylene and a graded ethanol series. After antigen retrieval, the sections were blocked with 3% hydrogen peroxide solution at room temperature for 15 minutes, followed by incubation with 3% BSA solution at room temperature for 30 minutes. After removing the blocking solution, CTSK antibody (1:3000, Proteintech Biotechnology, 11239-1-ap) was applied, and the sections were incubated overnight at 4 °C. After 3 washes with PBST, HRP-Polymer secondary antibody (Aifang Biotechnology, Changsha) was applied and incubated at room temperature for 30 minutes. Fluorescence development was performed using 520-TSA dye (Aifang Biotechnology, Changsha) for 5 minutes. The sections were mounted with DAPI-containing antifade mounting medium (Solarbio, Beijing), pressed overnight, and observed and imaged under an upright fluorescence microscope (Leica, DM4B).

### RT-qPCR reverse transcription quantitative polymerase chain reaction

The study complies with the *Declaration of Helsinki*. This study is a non-interventional one, and the collection of gingival crevicular fluid (GCF) was conducted during the patients' routine clinical treatment, without any intervention in their treatment plans or lifestyle, and without posing additional risks or discomfort. All patients have signed informed consent forms agreeing to the use of their GCF for research purposes. Moreover, the collected samples and data will be anonymized during the research process, ensuring no privacy breaches. Therefore, this study does not involve ethical risks, and according to relevant regulations, it does not require approval from the Ethics Committee of Xiangya Hospital, Central South University.

Six patients with normal gingiva and 6 patients with gingival recession who underwent straight-wire orthodontic treatment at Xiangya Hospital, Central South University, were selected. The gender and age information of the patients are shown in Table 1. GCF was collected by gently inserting dental absorbent paper points into the gingival sulcus of the lower anterior teeth for 30 seconds. The paper points were immediately placed into sterile centrifuge tubes containing 1 mL of Trizol, and RNA extraction and purification were performed according to the Trizol kit instructions. The RNA was finally dissolved in RNase-free water. The reverse transcription system was prepared using 5X Evo M-MLV RT Master Mix to reverse transcribe the RNA into cDNA. Quantitative amplification of the target gene was performed on a qPCR instrument using 2X SYBR Green Pro Taq HS Premix and specific primers. Gene expression levels were quantified using the 2−ΔΔCt method, with normalization to GAPDH. The primer sequences used in the study are listed below. GAPDH:Forward 5’—3’ AGGTCGGAGTCAACGGATTT, Reverse 5’—3’ GCCATGGGTGGAATCATATTGG; CTSK:Forward 5’—3’ GTTCTGCTGCTACCTGTGGT, Reverse 5’—3’ CCGAGAGATTTCATCCACCTTGT.

**Table 1:** Information on research subjects.

Healthy gingival group		Gingival recession group	
Gender	Age	Gender	Age
Female	20	Female	25
Female	23	Female	18
Female	22	Female	20
Male	22	Male	21
Male	19	Male	21
Male	25	Male	25

### Data analysis and statistics

Image analysis was performed using Image J software (National Institutes of Health, Bethesda, MD, USA), and statistical analysis and data visualization were conducted using GraphPad Prism software (version 10.0, GraphPad Software, San Diego, CA, USA). A two-sample t-test and 1-way ANOVA test was used to evaluate the differences in the data, with statistical significance set at *P* < .05.

## Results

### Single-cell RNA-seq and RNA-seq reveal the expression characteristics of CTSK in periodontitis gingival tissue

Single-cell RNA sequencing of gingival tissue from mice with early periodontitis (GSE201108) revealed that fibroblasts as the primary cell type expressing Ctsk in early periodontitis gingival tissue, with highly specific expression. The expression level of Ctsk in the fibroblast population was significantly higher than in other cell populations, and there was almost no detectable Ctsk expression in other cell types (Figure 1A-C).

RNA-seq analysis of healthy gingival tissue and periodontitis gingival tissue in mice (GSE186882) showed differences in gene expression between the two tissue samples. The expression levels of matrix metalloproteinase (MMP) family genes were significantly higher in periodontitis gingival tissue compared to healthy tissue. Specifically, Ctsk was highly expressed in the periodontitis group, with a significant upregulation compared to the healthy group (Figure 1D). GO enrichment analysis indicated significantly enrichment of Ctsk in biological processes such as "collagen metabolic process" (GO: 0032963), "collagen catabolic process" (GO: 0030574), and "tissue remodelling" (GO: 0048771) in periodontitis tissue (Figure 1E).

Analysis of single-cell RNA sequencing data from gingival tissue samples of healthy individuals and periodontitis patients (GSE152042) revealed that, consistent with the results from single-cell RNA sequencing of gingival tissue from mice with early periodontitis, CTSK expression in human gingival tissue was also highly specific to fibroblasts (Figure 1F-G). Moreover, although the number of fibroblasts in periodontitis gingival tissue was reduced compared to healthy tissue, the proportion of CTSK-positive fibroblasts increased (Figure 1H-I).

### Effects of orthodontic force on gingival height and thickness in the mouse OTM model

We established a mouse model of OTM on the maxillary first molar. Compared with the control group (CON), both OTM experimental groups showed a reduction in gingival height and thickness (Figure 2B). While the light-force group showed no statistically significant differences, the high-force group showed a significant reduction in mesial gingival height and thickness **(**Figure 2D).

### CTSK expression in gingival tissue during OTM gingival recession

**Fig. 3:**
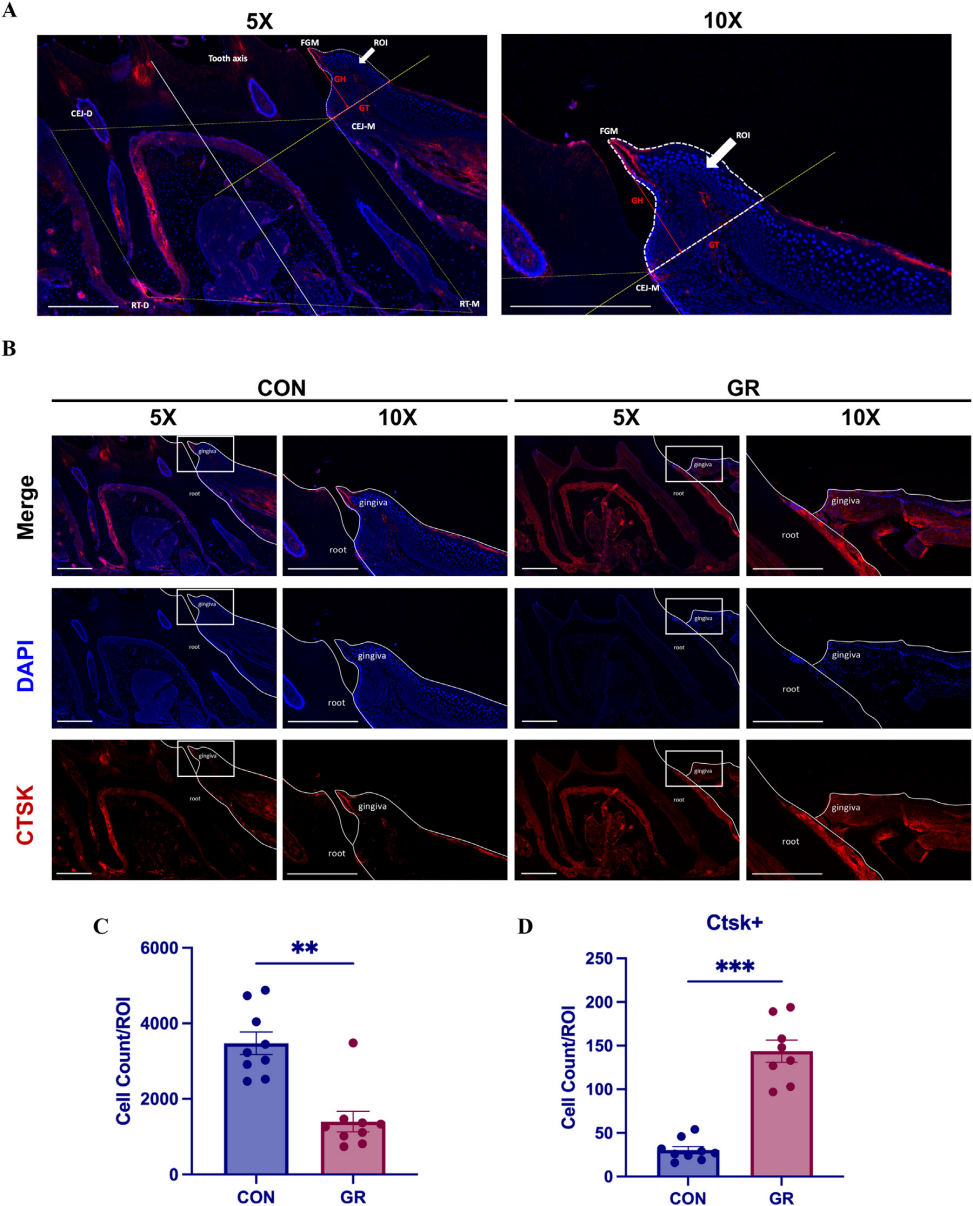
A, Schematic of the region of interest (ROI, indicated by the white dashed box) in the mesial gingival tissue of the first molar in mice; B, Immunofluorescence staining of CTSK in gingival tissues of the control (CON) and gingival recession (GR) groups; C, Statistical analysis of cell counts within the ROI of the mesial gingival tissue of the first molar in the control (CON) and gingival recession (GR) groups was performed using a two-sample t test (n = 9/group, **P < .01); D, Statistical analysis of CTSK+ cell counts within the ROI of the mesial gingival tissue of the first molar in the control(CON) and gingival recession (GR) groups was performed using a two-sample t test (n = 9/group, ***P < .001).

To explore the expression of CTSK during the process of gingival recession, we performed CTSK immunofluorescence staining to analyse the region of interest (ROI) in the mesial gingival tissue of the maxillary first molar in the mouse model of gingival recession induced by OTM under high force (Figure 3A). Cell count and CTSK-positive cell counting results revealed that compared to the control group (CON), the number of cells within the ROI in the gingival recession group (GR) had decreased, while the number of CTSK-positive cells in the same region had increased, with statistically significant differences (Figure 3B-D).

### Effect of CTSK inhibition on OTM gingival recession

**Fig. 4:**
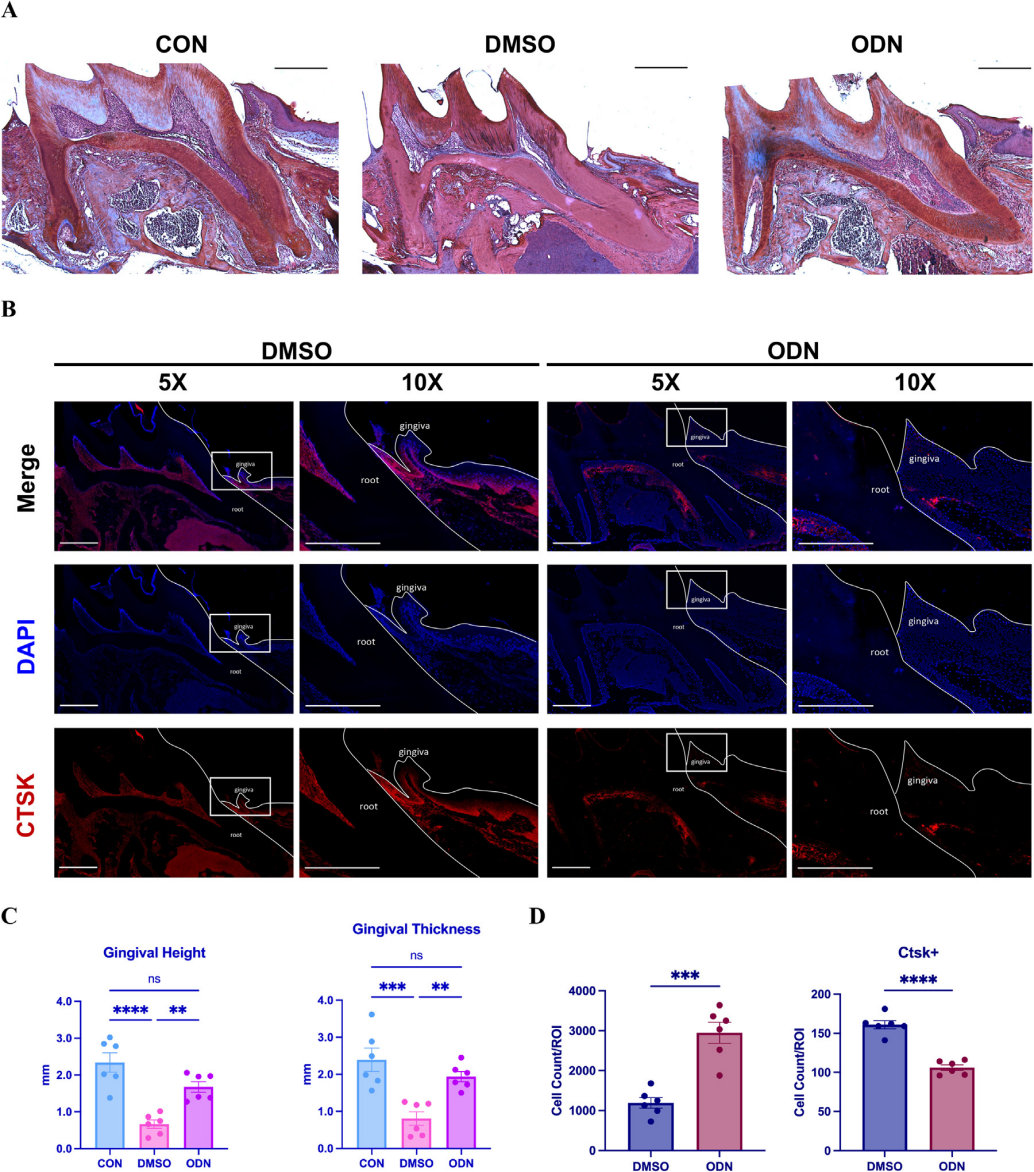
A, Gingival morphology of the control group (CON) and experimental group (DMSO and ODN); B, Immunofluorescence staining of CTSK in gingival tissues of the DMSO and ODN groups; C, Statistical analysis of gingival height (GH) and gingival thickness (GT) in the mesial gingival region of the first molar among the control group (CON) and experimental groups (DMSO and ODN) was performed using a 1-way ANOVA test (n = 6/group, **P < .01, ***P < .001, ****P < .0001); D, Statistical analysis of cell counts and CTSK+ cell counts within the ROI of the mesial gingival tissue of the first molar in the DMSO group and the ODN group was performed using a two-sample t test (n = 6/group, ***P < .001, ****P < .0001).

To further investigate the role of CTSK in gingival recession, we utilized the CTSK inhibitor ODN. Compared with the control group (CON), both OTM experimental groups showed a reduction in gingival height and gingival thickness (Figure 4A). In the DMSO group, the mesial gingival height and gingival thickness of the maxillary first molar showed a significant decrease compared to the control group (CON). However, in the ODN group, the reduction in gingival height and thickness was not statistically significant (Fig 4C).

Immunofluorescence staining showed that in the ROI of the mesial gingival tissue, the number of CTSK+ cells was lower in the ODN group than in the DMSO group, whereas the total cell count was higher, with statistically significant differences (Figure 4B and D).

### CTSK mRNA expression in GCF of orthodontic patients with gingival recession

**Fig. 5:**
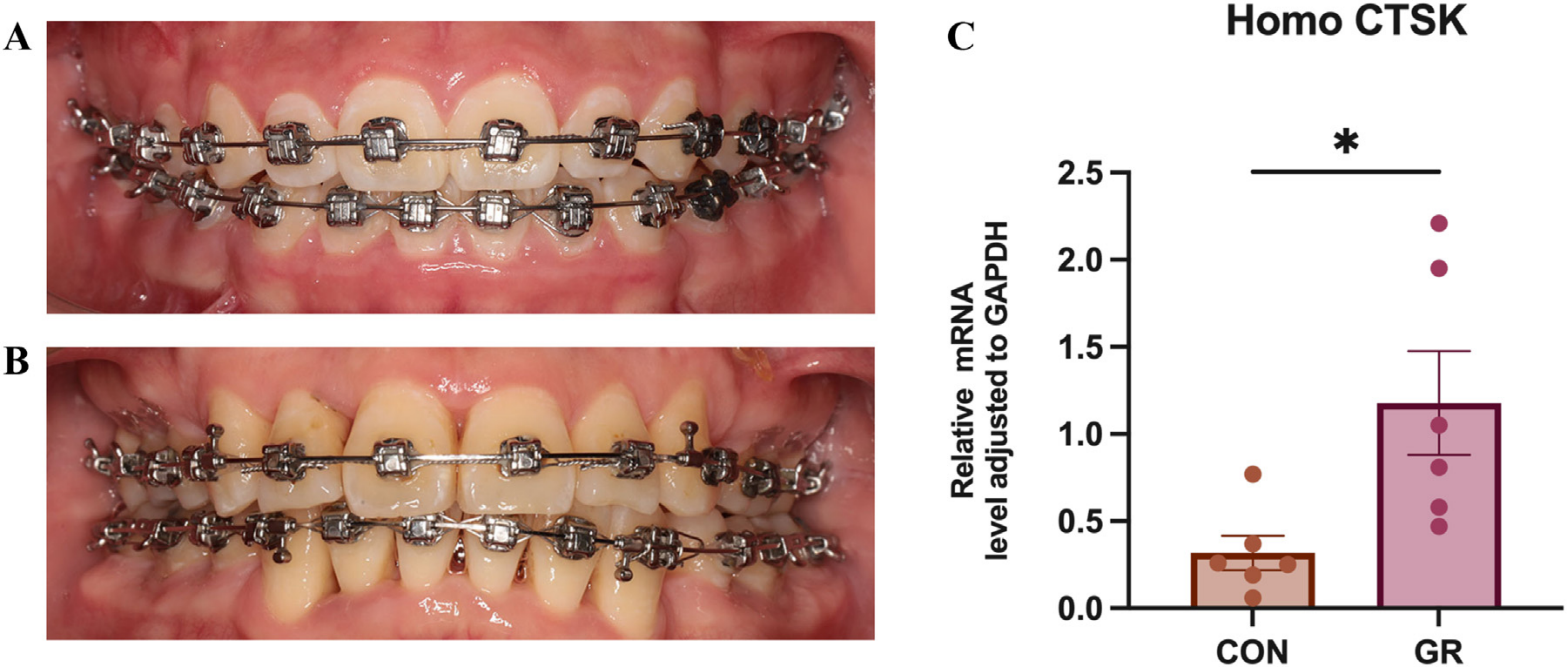
A, Intraoral image of the healthy gingival tissue group; B, Intraoral image of the gingival recession group; C, Statistical analysis of CTSK mRNA expression levels in the gingival crevicular fluid between orthodontic patients in the healthy gingival tissue group (CON) and the gingival recession group (GR) was performed using a two-sample t test (n = 6/group, *P < .05).

This study evaluated the expression levels of CTSK mRNA in the GCF of patients with healthy gingival tissue (CON) and those with gingival recession (GR) undergoing orthodontic treatment. Quantitative RT-qPCR analysis revealed significant differences in the expression of the CTSK mRNA between the two groups (*P* < .05), with significantly higher levels of CTSK mRNA in the GCF of patients with gingival recession compared to those with healthy gingival tissue (Figure 5C).

## Discussion

“Recession” refers to the reduction in the volume and number of cells in specific organs or tissues caused by sublethal cellular damage.^15^

Our animal experiments have shown that excessive orthodontic force contributes to gingival recession. Specifically, while light-force application did not induce significant changes in gingival height or thickness, high-force application resulted in a significant reduction in both parameters. This is consistent with previous studies indicating that high orthodontic forces can exacerbate soft tissue damage.^5^ Therefore, controlling orthodontic force magnitude is critical to minimizing gingival recession and maintaining periodontal health during treatment.

Cathepsin K (CTSK) is one of the most potent proteases in the lysosomal cysteine protease family, with its primary function being the mediation of bone resorption.^16^ An increasing number of studies indicate CTSK's unique and powerful role as a key marker of collagen degradation beyond bone tissue, particularly in fibroblast-mediated progresses. In addition to its well-established involvement in bone remodelling, CTSK has been shown to play a significant role in collagen degradation in tissues including the airways, lungs, joints, myocardium, and skin.^15^^,^^17^, ^18^, ^19^, ^20^, ^21^ Studies on the molecular mechanisms of periodontitis have found that elevated concentrations of CTSK in GCF are positively correlated with the severity of periodontitis.^22^^,^^23^ Recent experiments by Takeru Kondo et al on mice demonstrated that, in the early stages of periodontitis, gingival fibroblasts secrete CTSK into the connective tissue interstice, directly causing the degradation of connective tissue.^10^ Our analysis of single-cell RNA sequencing and RNA sequencing data from gingival tissues of mice and humans with periodontitis revealed high expression of CTSK in fibroblasts, further supporting its critical role in the degradation of gingival connective tissue (Figure 1). These findings provide strong evidence that CTSK can serve as a molecular marker for collagen fibre degradation. Given the specific high expression of CTSK in fibroblasts during the early stages of periodontitis, we hypothesize that gingival recession during OTM may also be closely associated with the upregulation of CTSK.

To validate this hypothesis, we used the previously established mouse model of gingival recession at the maxillary first molar to conduct immunofluorescence staining analyses of the gingival tissues to assess CTSK expression. Experimental results revealed a reduction in the number of cells within the ROI of the gingival tissue, while the number of CTSK-positive cells significantly increased. This finding is consistent with the results from single-cell RNA sequencing analysis of human periodontitis gingival tissues (Figure 1F-I), suggesting that CTSK may be directly related to gingival tissue recession during OTM. Moreover, ODN, as a CTSK inhibitor, effectively reduces the number of CTSK+ cells in gingival tissue and partially mitigates the decrease in gingival height and thickness. These findings suggest that CTSK, as a protease capable of degrading the extracellular matrix, promotes the degradation of the extracellular matrix of gingival connective tissue, leading to tissue damage and a reduction in cell count, ultimately resulting in reduced gingival height and thickness, manifested as gingival recession. Inhibition of CTSK expression may help alleviate high-force OTM induced gingival recession, suggesting that ODN and other CTSK inhibitors may serve as potential therapeutic strategies for gingival recession.

GCF is known to contain not only proteins but also a diverse population of cells, including desquamated epithelial cells, cytokines, electrolytes, and bacteria from adjacent plaques.^24^^,^^25^ Given the presence of cellular components in GCF, we performed RT-qPCR analysis on samples from orthodontically treated patients, which revealed significantly higher CTSK gene expression in the gingival recession group compared to the control group. As GCF may be recognized as a new source of diagnostic and prognostic biomarkers of oral diseases, this finding further supports the critical role of CTSK in the pathological process of OTM gingival recession. The cellular components within the gingival tissue may contribute to the degradation of gingival connective tissue by upregulating CTSK expression.

It is noteworthy that although animal experiments have reported incidents of gingival recession following orthodontic treatment,^26^, ^27^, ^28^ and clinical studies have reported a significantly higher incidence of gingival recession in patients after orthodontic treatment compared to before treatment,^29^ it is generally believed that gingival recession is not a direct result of orthodontic treatment. Research has shown that compared to a thick gingival biotype, a thin gingival biotype is more susceptible to gingival recession following orthodontic treatment.^30^, ^31^, ^32^, ^33^ Additionally, the use of fixed orthodontic appliances can increase the risk of gingival inflammation due to plaque accumulation, thereby increasing the likelihood of gingival recession.^30^^,^^34^ Studies have also found an association between over 10 degrees of anterior tipping of the mandibular incisors and the occurrence of gingival recession,^35^ with each additional degree of tooth proclination increasing gingival recession by about 0.2 mm.^36^ However, more studies suggest that the degree of anterior tipping of mandibular incisors is not directly related to the occurrence of gingival recession.^7^^,^^37^, ^38^, ^39^ Therefore, gingival recession following orthodontic treatment may result from a combination of factors including gingival biotype, inflammation, soft tissue thickness, and orthodontic forces.

This study has some limitations. First, the extrapolation of animal experiment results should be approached with caution. Future studies should include more clinical cases and measure CTSK protein levels using methods such as ELISA to enhance the clinical relevance of the findings and more comprehensively assess the role of CTSK in gingival recession. Secondly, it is necessary to include different time points to more comprehensively analyse the dynamic process of gingival recession. Thirdly, Finally, further in-depth studies are needed to explore which cellular components, such as gingival fibroblasts, participate in the regulation of CTSK expression in order to more fully reveal its molecular mechanisms in the process of gingival recession.

In summary, this study is the first to identify CTSK as a marker of collagen degradation in gingival recession induced by high-force orthodontic tooth movement, thereby providing a new target for exploring the molecular mechanisms of gingival recession. CTSK inhibitors, such as ODN, partially mitigates the decrease in gingival height and thickness. These findings may offer new insights for the clinical management of complications such as "black triangles" following orthodontic treatment.

## Conflict of interest

None disclosed.
